# A computational model study of the influence of the anatomy of the circle of willis on cerebral hyperperfusion following carotid artery surgery

**DOI:** 10.1186/1475-925X-10-84

**Published:** 2011-09-23

**Authors:** Fuyou Liang, Kazuaki Fukasaku, Hao Liu, Shu Takagi

**Affiliations:** 1 Computational Science Research Program, RIKEN, Wako, Saitama, Japan; 2 Department of Neurosurgery, Himonya hospital, Tokyo, Japan; 3 Graduate School of Engineering, Chiba University, Chiba-Shi, Chiba, Japan; 4 Department of Mechanical Engineering, The University of Tokyo, Tokyo, Japan

## Abstract

**Background:**

Cerebral hyperperfusion syndrome develops in a small subset of patients following carotid artery surgery (CAS) performed to treat severe carotid artery stenosis. This syndrome has been found to have a close correlation with cerebral hyperperfusion occurring after CAS. The purpose of this study is to investigate whether and how the anatomy of the Circle of Willis (CoW) of the cerebral circulation influences post-CAS cerebral hyperperfusion.

**Methods:**

A computational model of the cerebral circulation coupled with the global cardiovascular system has been developed to investigate hemodynamic events associated with CAS. Nine topological structures of the CoW were investigated in combination with various distribution patterns of stenosis in the feeding arteries of the cerebral circulation.

**Results:**

The occurrence of post-CAS cerebral hyperperfusion was predicted for the CoW structures that have poor collateral pathways between the stenosed cerebral feeding arteries and the remaining normal feeding arteries. The risk and the localization of post-CAS hyperperfusion were determined jointly by the anatomy of the CoW and the distribution pattern of stenosis in the cerebral feeding arteries. The presence of basilar artery stenosis or contralateral ICA stenosis increased the risk of post-CAS hyperperfusion and enlarged the cerebral region affected by hyperperfusion. For a certain CoW structure, the diameters of the cerebral communicating arteries and the severity of carotid artery stenosis both had a significant influence on the computed post-CAS cerebral hyperperfusion rates. Moreover, post-CAS cerebral hyperperfusion was predicted to be accompanied with an excessively high capillary transmural pressure.

**Conclusions:**

This study demonstrated the importance of considering the anatomy of the CoW in assessing the risk of post-CAS cerebral hyperperfusion. Particularly, since the anatomy of the CoW and the distribution pattern of stenosis in the cerebral feeding arteries jointly determine the risk and localization of post-CAS cerebral hyperperfusion, a patient-specific hemodynamic analysis aimed to help physicians identify patients at high risk of cerebral hyperperfusion should account for the combined effect of the anatomy of cerebral arteries and cerebral feeding artery stenoses on cerebral hemodynamics.

## Background

Extracranial internal carotid artery (ICA) stenosis accounts for 15-20% of ischemic strokes and is usually treated by carotid artery surgery (CAS) such as carotid endarterectomy or stenting [1,2]. A potential risky problem with CAS is that cerebral hyperperfusion syndrome (CHS) (characterized by ipsilateral headache, seizure or intracranial hemorrhage (ICH)) develops in a small subset (0.75-3%) of patients following successful CAS [2]. Although rare, CHS can lead to significant morbidity and mortality if not correctly recognized and treated [2,3].

The most pronounced hemodynamic event associated with CAS is a sudden increase in cerebral blood flow (CBF). Generally, an over 100% increase in CBF after CAS compared to the pre-CAS value is considered as hyperperfusion [4]. Post-CAS hyperperfusion has been observed in 9-14% of patients in clinical studies [2,4] and suggested to be an important hemodynamic factor underlying CHS, for instance, the risk of developing CHS is 10 times higher in patients with hyperperfusion than those without [2], and ICH develops in 3.3% of patients with hyperperfusion vs. only 0.24% of those without [4]. In fact, there is evidence that identifying patients at high risk of hyperperfusion and treating them early help to reduce the incidence of ICH and lead to better prognosis [5,6].

Clinical studies [2,7] have identified some risk factors for CHS or cerebral hyperperfusion, such as hypertension, high-grade ICA stenosis, decreased cerebral vasoreactivity and contralateral ICA stenosis. Most of these factors are associated closely with hemodynamics in the cerebral circulation. In fact, emerging evidence supports that a pre-operative evaluation of cerebral hemodynamic status may help to identify patients at high risk of post-operative hyperperfusion [5,6,8]. The cerebral circulation possesses many collateral vessels which play an important role in maintaining cerebral perfusion in case occlusive disease develops in the feeding arteries of the cerebral circulation [9]. Cerebral collateral vessels are commonly divided into primary and secondary collateral pathways, with the former constituted mainly by the Circle of Willis (CoW), while the latter by the ophthalmic artery and leptomeningeal vessels [9]. Many studies have demonstrated that the status of primary collateral flows is a determinant factor for clinical symptoms and outcomes of intervention in patients with severe ICA stenosis [10-12]. Although the secondary collaterals may also play some roles in compensating for severe ischemia [13,14], their compensatory capability seems to be limited [15].

Theoretically, a complete CoW is able to maintain sufficient cerebral perfusion when any single cerebral feeding artery is occluded. However, this ability can be impaired by a topological variation in CoW and coexistence of stenoses in multiple feeding arteries. In fact, a complete CoW structure exists in only about 50% of the population, with various incomplete CoW structures existing in the remaining population [16,17]. The role of the anatomy of the CoW in regulating cerebral blood flows has been well described [17]; whereas, it remains unclear how the anatomy of the CoW influences post-CAS hyperperfusion, particularly when occlusive disease is present in multiple cerebral feeding arteries. To answer this question, we have developed a novel computational model of the cerebral circulation which is capable of describing cerebral hemodynamics under various physiological/pathological conditions.

## Methods

### Cardiovascular model

**Figure 1:**
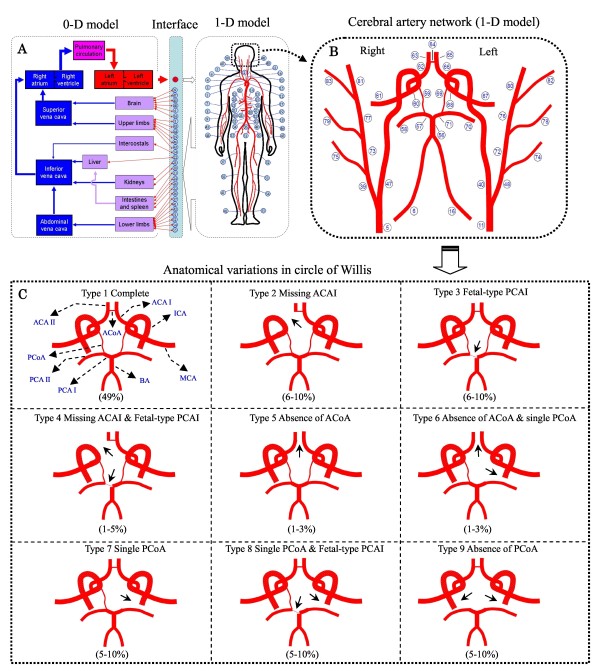
**Schematic description of a computational model of the cardiovascular system (Panel A)**. The cerebral circulation is modeled as a portion of the cardiovascular system (Panel B). The cerebral feeding arteries are No. 40/47 - left/right ICA and No. 56 - Basilar artery (BA), communicating arteries are No. 59/69 - left/right posterior communicating artery (PCoA) and No. 64 - anterior communicating artery (ACoA), and cerebral efferent arteries are No. 58/70 - the right/left posterior cerebral artery II (PCA II), No. 61/67 - right/left middle cerebral artery (MCA) and No. 63/65 - right/left anterior cerebral artery II (ACA II). Panel C illustrates the nine typical CoW structures. The value given under each structure indicates the appearance frequency of the structure in the population. The arrows denote the locations of missing or fetal-type arteries.

**Figure 2:**
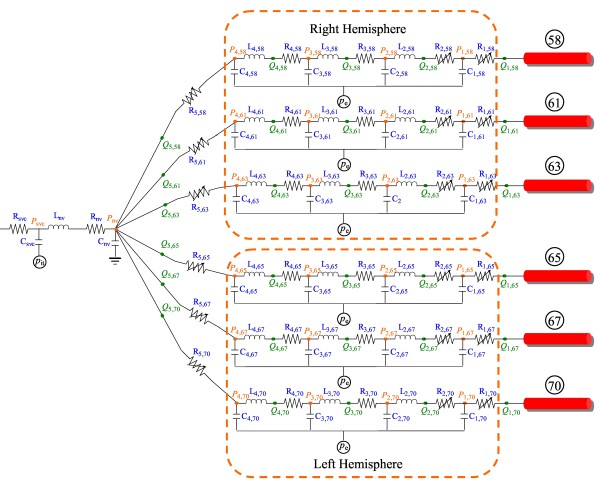
**Lumped parameter modeling of the distal vascular systems of the intracranial cerebral circulation**. The vascular systems distal to each cerebral efferent artery are divided into four compartments (namely, arteriolar, capillary, venular and venous compartments) with each compartment represented by a certain assembly of lumped parameters. The flows through all the distal vasculatures are assumed to converge into the neck veins. Please see the text for more details.

A cardiovascular model developed in our previous studies [18] has been extended to include the cerebral circulation (see Figure 1(A, B)). The cardiovascular model describes pulse wave propagation in the largest 83 arteries with a one-dimensional (1-D) sub-model while describing hemodynamics in the remaining cardiovascular system (including the microcirculation, the pulmonary circulation and the heart) with a lumped parameter (0-D) sub-model. The intracranial cerebral artery network is comprised by the 18 largest arteries (see Figure 1(B)) and the downstream vascular system corresponding to each cerebral efferent artery is divided into arteriolar, capillary, venular and venous compartments (see Figure 2). To reduce the degree of the complexity of the cerebral circulation model, we have neglected the smaller cerebral arteries and the secondary collateral vessels. Cerebral venous flows in different cerebral regions were assumed to converge to the neck veins. An extravascular pressure was applied to the intracranial vessels to account for the critical closing pressure (*p*_e _in Figure 2) at which blood flow to the brain stops. In addition, the cerebral arteriolar resistances and venous resistances were set to be changeable with cerebral hemodynamic conditions. These characteristics enabled the model to simulate cerebral hemodynamics under various physiological/pathological conditions.

#### 1-D governing equations for pulse wave propagation in the arteries

Blood flows in an artery were described by the 1-D mass and momentum conservation equations derived by integrating the three-dimensional mass conservation and Navier-Stokes equations over the cross-section of the artery [19].

(1) $$\frac{\partial A}{\partial t}+\frac{\partial AU}{\partial z}=0,$$

(2) $$\frac{\partial U}{\partial t}+\frac{\partial }{\partial z}\left(\frac{U^{2}}{2}+\frac{P}{\rho }\right)=K_{\upsilon }\frac{U}{A}$$

The system of Eqs. 1 and 2 was completed by a pressure-area relationship that has been previously used to describe the deforming response of arterial wall to changes in transmural pressure [17-23].

(3) $$P=P_{0}+P_{\text{e}}+\beta \left(\sqrt{A}-\sqrt{A_{0}}\right),\;\text{with}\;\beta =\frac{\sqrt{\pi }Eh_{0}}{r_{0}\left(1-\sigma^{2}\right)}.$$

Here, *t *is the time, *z *the axial coordinate along the artery; and *ρ *the blood density (*ρ *≈ 1.06 g/cm^3^); *A, U *and *P *represent the lumen area, mean flow velocity and intravascular blood pressure, respectively; and *K*_υ _is the coefficient of the viscous term; *P*_0 _is the reference pressure at *A = A*_0 _and was set to be 85 mmHg; *P*_e _is the extravascular pressure; *E *is the Young's modulus; *h*_0 _the wall thickness; *r*_0 _the radius of the artery at the reference pressure; and *σ *the Poisson's ratio, here taken to be 0.5 by assuming arterial wall to be incompressible.

It is noted that, the cross-sectional velocity profile of blood flow changes transiently over a cardiac cycle and varies along the arterial system; this raises an issue as to how to correctly model the convective and viscous terms when reducing a three-dimensional blood flow model into a 1-D model. At this point, many modeling methods have been proposed based on various assumptions [17,18,20,24-27]. In this study, we employed a relatively simple modeling method in which the coefficient of the viscous term in Eq.2 is taken to be -8π*υ (υ *being the kinematic viscosity of blood ≈ 0.045 cm^2^/s) based on a Poiseuille flow assumption [20,21], while the 1-D convective term is derived by assuming a flat velocity profile [17,19-22]. These assumptions led to several simplifications in the numerical treatment of flow conditions at the boundaries [19,21]. Meanwhile, the error induced by the assumptions in the prediction of blood flow distribution in the cardiovascular system should be negligible since normal large arteries generate fairly less blood pressure loss in comparison with the downstream resistant micro-vasculatures [18].

From Eq. 3, arterial transmural pressure is related linearly to the change in arterial radius relative to its reference value. According to the data reported in previous experimental studies [28,29], the linear relation is acceptable when arterial transmural pressure varies within the physiological range (e.g., from diastolic to systolic pressure). Previous computational studies [17-23] have indeed demonstrated that employing the relation does not prevent a reasonable prediction of pulse wave propagation in large arteries. However, it should be noted that a linear pressure-radius relation fails to be proper when arterial transmural pressure varies beyond the general physiological range. Experimental studies [30] have demonstrated that when transmural pressure is reduced progressively from an over systolic to minus value, the pattern of arterial wall deformation changes from stretching to buckling and collapsing, exhibiting a highly non-linear pressure-radius relation.

Flows in different arteries were linked by imposing the conservation of mass and continuity of total pressure at the bifurcations [17-23].

#### Stenosis model

Eq. 2 cannot fully account for the pressure drop induced by an arterial stenosis. To compensate for this limitation, an experiment-based empirical stenosis model [31] has been incorporated to relate the stenosis-induced pressure drop to the geometry of stenosis:

(4) $$\Delta P=\frac{K_{\text{v}}\mu }{A_{0}D_{0}}Q+\frac{K_{\text{t}}\rho }{2A_{0}^{2}}{\left(\frac{A_{0}}{A_{\text{s}}}-1\right)}^{2}Q\left|Q\right|+\frac{K_{\text{u}}\rho L_{\text{s}}}{A_{0}}\overset{{}^{\circ}}{Q},$$

where Δ*P *and *Q *denote pressure drop and flow rate through the stenosis, respectively; *Q *is the time derivative of *Q, A*_0 _and *A*_s _refer to the cross-sectional areas of the normal and stenotic segments, respectively, *L*_s _represents the stenosis length, and * μ*is the blood viscosity. Further, *K*_v_, *K*_t _and *K*_u _are empirical coefficients, with *K*_v _= 32(0.83*L*_s _+ 1.64*D*_s_)×(*A*_0_/*A*_s_)^2^/*D*_0_, *K*_t _= 1.52, and *K*_u _= 1.2, where *D*_0 _and *D*_s _are the diameters corresponding to *A*_0 _and *A*_s_. The degree (severity) of stenosis is defined as the percentage reduction in arterial diameter (= (1-*D*_s_/*D*_0_) ×100%).

#### Governing equations for the 0-D sub-model of the cerebral circulation

Following the general 0-D modeling method [19,32,33], the viscous resistance, blood inertia and compliance of each vascular segment were mimicked respectively by three electric components (resistor (*R*), inductor (*L*) and capacitor (*C*)). In analogy to the principles of electric circuit, the governing equations were formulated by imposing mass and momentum conservation along the flow pathway (from arterioles to veins) (see Figure 2).

**Figure 3:**
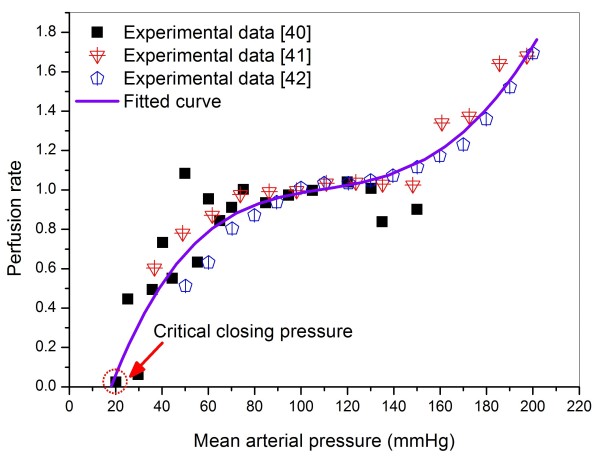
**Relationship between cerebral perfusion pressure and perfusion rate**. The continuous line represents the fitted fourth-degree polynomial function based on the experimental data.

At a 'capacitor' component, mass conservation reads

(5) $$\frac{\text{d}V_{i,j}}{\text{d}t}=Q_{i,j}-Q_{i+1,j},$$

and at an 'inductor' component, momentum conservation reads

(6) $$\frac{\text{d}Q_{i,j}}{\text{d}t}=\frac{P_{i-1,j}-Q_{i,j}R_{i,j}-P_{i,j}}{L_{i,j}},$$

where *V, Q *and *P *represent blood volume, flow rate and blood pressure, respectively; *P *is related to *V *by *P = V/C + P*_e_, with *P*_e _being the critical closing pressure, here taken to be 18.1 mmHg in accord with the cerebral autoregulation curve (see Figure 3). The subscript '*i*' is a sequence number that is increased from the arterial side toward the venous side (*i *∈ *I *= [1,4]); whereas '*j*' denotes the labeled number of the cerebral efferent arteries in the 1-D sub-model (*j *∈ *J *= {58, 61, 63, 65, 67, 70}) (see Figure 2).

Specially, cerebral vein may collapse due to the effect of extravascular pressure (*P*_e_), resulting in a varying venous resistance. This has been accounted for by modeling cerebral venous resistance (*R*_5, *j *_in Figure 2) as a function of cerebral venous blood pressure (*P*_4, *j*_), downstream neck venous pressure (*P*_nv_) and extravascular pressure (*P*_e_) [34,35].

(7) $$R_{\text{5,}j}=R_{\text{v0,}j}\cdot \frac{P_{\text{4,}j}-P_{\text{nv}}}{P_{\text{4,}j}-P_{\text{e}}},$$

where *R*_v0,*j *_is a constant venous resistance component.

Cerebral venous flow (*Q*_5, *j *_in Figure 2) can be calculated as

(8) $$Q_{\text{5,}j}=\frac{P_{\text{4,}j}-P_{\text{nv}}}{R_{\text{5,}j}}.$$

Substituting Eq. 7 into Eq. 8, one gets

(9) $$Q_{\text{5,}j}=\frac{P_{\text{4,}j}-P_{\text{e}}}{R_{\text{v0,}j}}.$$

From Eq. 9, cerebral venous flow is independent of the extracranial venous pressure (*P*_nv_) as far as *P*_e _is larger than *P*_nv _[36].

0-D modeling of other portions of the cardiovascular system, such as the pulmonary circulation, the heart, has been described in detail in [18,19].

#### Numerical methods

The equations system of the cardiovascular model consists of a 1D partial differential-algebraic sub-system coupled with a 0D ordinary differential-algebraic sub-system. The two sub-systems were solved numerically using the two-step Lax-Wendroff method and a fourth-order Runge-Kutta method, respectively. The solutions of the sub-systems were then linked at the 0-1D interfaces where mass and momentum conservation is imposed. More details on the numerical methods employed to treat flow conditions at the bifurcations and the 0-1D interfaces have been given elsewhere [19].

### Cerebral autoregulation

Generally, cerebral autoregulation is a dynamic process, for instance, it takes several to several tens of seconds to restore cerebral perfusion upon an abrupt change in perfusion pressure [37-39]. For the present problem, since a chronic ICA stenotic disease imposes a long-term influence on cerebral perfusion, a static cerebral pressure-perfusion rate relationship (herein termed cerebral autoregulation curve) would be sufficient, and which has herein been constructed by fitting a fourth-degree polynomial function to available experimental data [40-42] (see Figure 3). Note that the data points that deviate apparently from the neighboring data points have been considered as noise data and removed from the input data of function fitting. The perfusion rate represents the ratio of real flow rate to normal flow rate. The autoregulation curve was incorporated into the model by adjusting the cerebral arteriolar resistances via a negative feedback process.

(10) $$R^{n+1}=R^{n}\left(1-\alpha \frac{{\bar{Q}}^{\text{T}}-{\bar{Q}}^{n}}{{\bar{Q}}^{\text{T}}}\right)$$

Here *R *represents the arteriolar resistance corresponding to each cerebral efferent artery and *Q *the mean flow rate averaged over a cardiac cycle. Since resistance adjustment was performed at intervals of a cardiac cycle, the upper subscript '*n*' denotes current cardiac cycle, whereas '*n*+1' indicates the next cardiac cycle. *Q*^T ^denotes the target flow rate calculated from the cerebral autoregulation curve. *α *is the under-relaxation factor used to stabilize the numerical simulation (here taken to be 0.9).

### Anatomical variations in CoW

Based on the data collected from the literature [16,17,43,44], we categorized the frequently observed anatomical variations in CoW into nine types (see Figure 1(C)). Each type has a specific frequency of appearance in the population, with type 1 being the most prevalent structure.

### Physiological data

The geometrical parameters of the cerebral arteries were assigned based on the data reported in the literature [17,24,27] and the in vivo data available in our lab. The elastic parameters of the arteries have been estimated according to an elastic modulus-artery radius relationship constructed based on experimental data [26]. The reference flow rate through each cerebral efferent artery at normal perfusion pressure was assigned based on previously reported data [24,27,43,45], giving a flow division among PCA II, MCA and ACA II of 0.96:2:1 and a total cerebral flow rate of about 12 ml/s. The assigned data for the cerebral circulation model are summarized in Table 1. The data for other portions of the cardiovascular system have been given in our previous studies [18,19].

**Table 1:** Physiological data of the cerebral circulation

**No**.	Arterial segment	*L*[cm]	*r*_0_[cm]	*r*_1_[cm]	*c*_0_[m.s^-1^]	*R*_T _[mmHg.s. ml^-1^]
5	R. common carotid	17.7	0.400	0.370	5.92	-
11	L. common carotid	20.8	0.400	0.370	5.92	
6/16	R./L. vertebral	13.5	0.150	0.136	11.9	-
39/48	R./L. ext.carotid I	4.10	0.200	0.150	8.90	-
40/47	L./R. int. carotid I	17.6	0.250	0.200	7.90	-
56	Basilar	2.90	0.162	0.162	9.33	-
57/71	R./L. PCA I	0.50	0.107	0.107	12.93	-
58/70	R./L. PCA II	8.60	0.105	0.105	13.13	39.13
59/69	R./L. PCoA	1.50	0.073	0.073	17.24	-
60/68	R./L. int. carotid II	0.50	0.200	0.200	8.26	-
61/67	R./L. MCA	11.90	0.143	0.143	10.23	19.21
62/66	R./L. ACA I	1.20	0.117	0.117	12.03	-
63/65	R./L. ACA II	10.30	0.120	0.120	11.77	38.75
64	ACoA	0.3	0.100	0.100	17.08	-
72/73	L./R. ext.carotid II	6.10	0.200	0.200	8.53	-
74/75	L./R. sup. thy. asc. ph. lyng. fac. occ.	10.10	0.100	0.100	16.57	225.6
76/77	L./R. superf. temp.	6.10	0.160	0.160	9.62	-
78/79	L./R. maxillary	9.10	0.110	0.110	15.09	188.0
80/81	L./R. superf. temp. fron. bran.	10.0	0.110	0.110	15.09	188.0
82/83	L./R. superf. temp. pari. bran.	10.1	0.110	0.110	15.09	188.0

Here, *r*_0 _and *r*_1 _denote the proximal and distal radii of an arterial segment, *c*_0 _is the pulse wave velocity at the middle of each arterial segment at the reference state (*A*_0_, *U*_0_), *R*_T _is the total distal vascular resistance corresponding to each efferent artery.

### Computation conditions

Each set of computation comprised three steps, with the computation for each step being continuously run for 30 cardiac cycles to guarantee the convergence of computation (inter-cardiac cycle error for mean flow rate within 0.1%): during the first 30 cardiac cycles (Step I), the reference cerebral arteriolar resistances are estimated under normal perfusion conditions (in the absence of artery stenosis); during the second 30 cardiac cycles (Step II), stenoses are introduced in certain cerebral feeding arteries (including at least one or both of the ICA) and the cerebral arteriolar resistances are further modified to match the cerebral autoregulation curve; and at the beginning of the last 30 cardiac cycles (Step III), an ICA stenosis is suddenly removed to simulate CAS. The post-CAS hyperperfusion rate in each cerebral efferent artery is calculated at the end of step III. It is noted that we have herein assumed that cerebral arteriolar resistances do not change immediately after CAS in order to simulate the largest post-CAS hyperperfusion rate.

The nine types of CoW structure illustrated in Figure 1(C) were studied. Each type of CoW structure was further investigated in combination with three distribution patterns of stenosis in the cerebral feeding arteries: (1) unilateral ICA stenosis, (2) bilateral ICA stenosis, and (3) coexisting unilateral ICA stenosis and basilar artery (BA) stenosis. The degree of each stenosis was set uniformly to be 75% to represent a severe stenotic condition. Heart rate has been fixed at 60BPM in all the computations.

### Definition of hyperperfusion rate

Hyperperfusion rate (*C*_H_) was defined as the percentage change of post-CAS flow rate relative to the pre-CAS value [4].

(11) $$C_{H}=\left({\bar{Q}}_{\text{a}}∕{\bar{Q}}_{\text{b}}-1\right)\times 100\%$$

Here, *Q*_b _and *Q*_a _refer respectively to the mean flow rates before and after CAS.

## Results

### Computed flow rates through the left/right ICA and the BA for different CoW structures under normal conditions

The computed mean flow rates through the left/right ICA and the BA for three CoW structures (Types 1 to 3) under normal conditions (in the absence of feeding artery stenosis) are given in Table 2 in comparison with the corresponding in vivo data [43]. It was observed that the pattern of flow division among the three cerebral feeding arteries depends strongly on the CoW structure. And the computations reasonably captured the flow division patterns described by the in vivo data.

**Table 2:** Mean flow rates through the cerebral feeding arteries computed for three CoW structures under normal conditions (compared with measured data [43])

CoW structure	Computation			**Measurement **[43]		
	L. ICA	R. ICA	BA	L. ICA	R. ICA	BA
Type 1	4.81	4.85	2.36	5.07	5.18	2.75
Type 2	6.14	3.50	2.36	6.12	3.93	2.35
Type 3	4.80	5.70	1.49	5.28	5.91	1.50

All the values have a unit of ml/s.

### Hemodynamics before and after CAS

**Figure 4:**
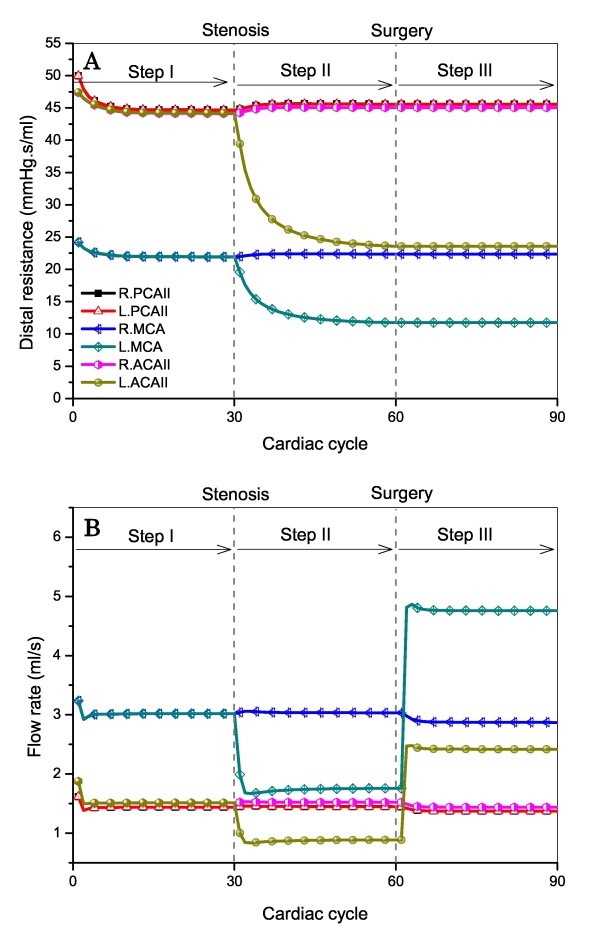
**Computed changes in distal resistances of (Panel A) and mean flow rates through (Panel B) the efferent arteries of the type 6 CoW structure before and after CAS (unilateral left ICA stenosis)**. In the absence of ICA stenosis, the distal resistance corresponding to each cerebral efferent artery is gradually changed from its initial value (roughly assigned at the beginning of computation) to a reference value (step I). The distal resistances of the left MCA and ACA II are further reduced by about 50% following the onset of the left ICA stenosis (step II). After CAS is implemented at the beginning of step III, marked flow overshoots through the left MCA and ACA II appear.

The type 6 CoW structure was taken as an example to illustrate the hemodynamic changes corresponding to onset of ICA stenosis and CAS. Herein, a stenosis was generated in the left ICA at the beginning of Step II and removed at the beginning of Step III. Before CAS, the cerebral distal resistance corresponding to each cerebral efferent artery was gradually modified from its initial value to regulate the flow rate toward the target value (see Figure 4). After CAS, the flow rates through the left MCA and ACA II were remarkably increased; whereas the flow rates through other cerebral efferent arteries were less changed. The computed hyperperfusion rates for the left MCA and ACA II were both larger than 100% (being 171% and 173%, respectively), indicating the occurrence of post-CAS hyperperfusion in the left MCA and ACA II territories.

**Figure 5:**
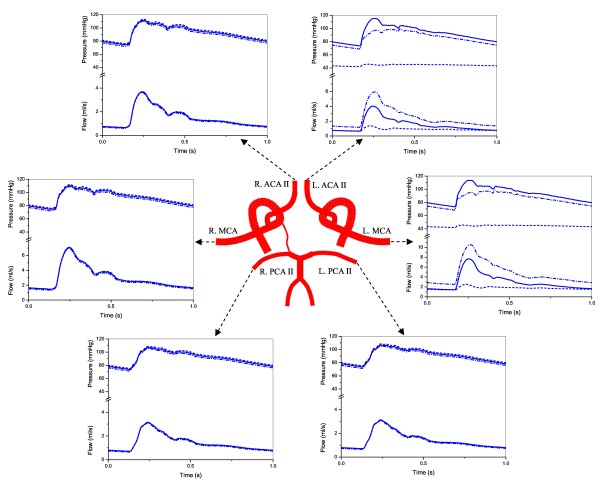
**Computed changes in pressure/flow waves in the efferent arteries of the type 6 CoW structure before and after CAS: continuous line (normal), dashed line (in the presence of left ICA stenosis), dash-dotted line (after removal of the stenosis)**.

The pre- and post-CAS blood flow/pressure waves in the six cerebral efferent arteries are plotted together with the results for normal conditions (in the absence of ICA stenosis) in Figure 5. As expected, the pressure/flow waves in the hyperperfusion-affected arteries (left MCA, ACA II) changed significantly with the onset and removal of the ICA stenosis. In contrast, the pressure/flow waves in the remaining cerebral efferent arteries showed little change.

**Figure 6:**
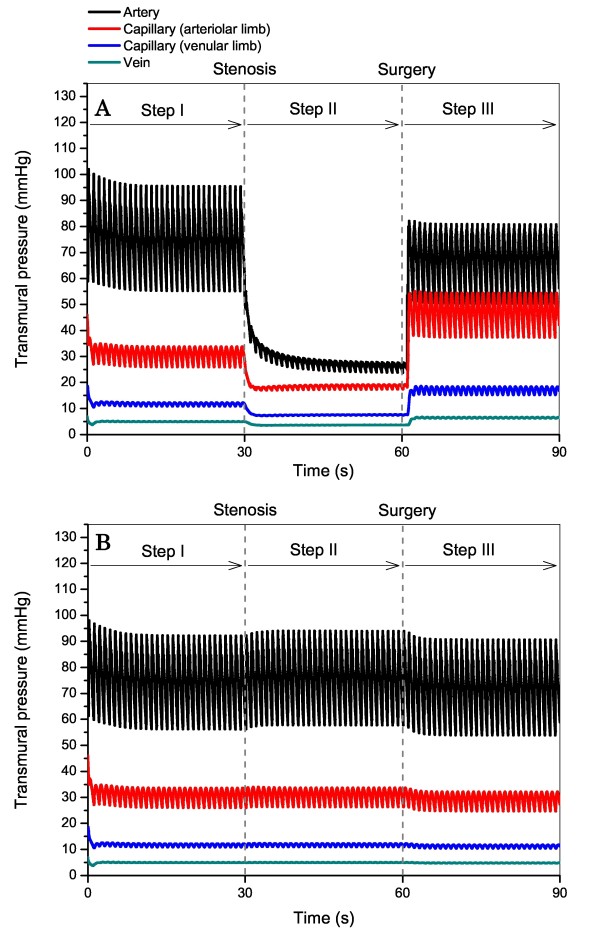
**Computed changes in transmural pressures distal to the left MCA (Panel A) and right MCA (Panel B) of the type 6 CoW structure before and after CAS (unilateral left ICA stenosis)**. The onset and removal of the left ICA stenosis induce pronounced changes in transmural pressures distal to the left MCA in which post-CAS hyperperfusion occurs, but have little influence on transmural pressures distal to the right MCA.

Figure 6 shows the transmural pressures distal to the left MCA (A) and right MCA (B). The transmural pressures distal to the left MCA were reduced as the ICA stenosis is present and increased after the stenosis is removed via CAS; whereas, those distal to the right MCA showed little change during the entire process.

### Hyperperfusion rates in the case of unilateral ICA stenosis

**Figure 7:**
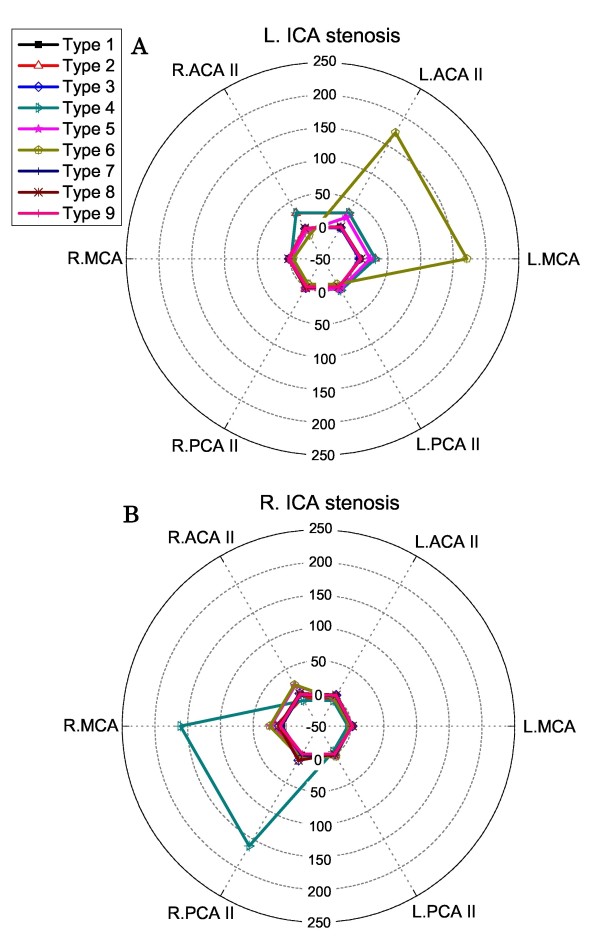
**Polar plots of the computed post-CAS hyperperfusion rates in the cerebral efferent arteries in the cases of unilateral left ICA stenosis (Panel A) and right ICA stenosis (Panel B)**.

Figure 7 shows the computed post-CAS hyperperfusion rates for the nine types of CoW structure in the cases of unilateral left ICA stenosis (A) and right ICA stenosis (B). The type 6 and type 4 CoW structures were found to induce hyperperfusion in the left ICA stenosis case and the right ICA stenosis case, respectively.

### Hyperperfusion rates in the case of bilateral ICA stenosis

**Figure 8:**
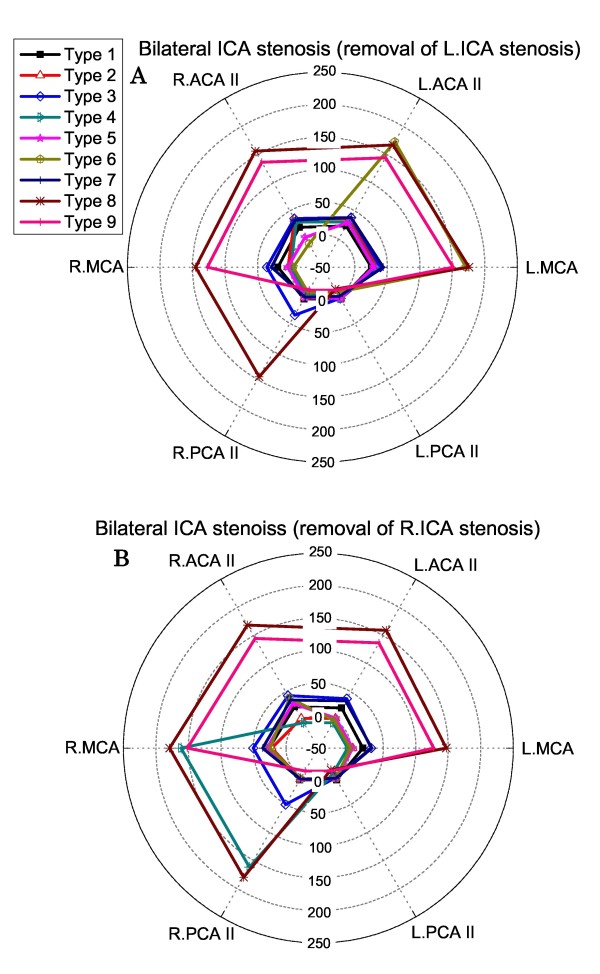
**Polar plots of the computed post-CAS hyperperfusion rates in the cerebral efferent arteries in the case of bilateral ICA stenosis: removal of the left ICA stenosis (Panel A), removal of the right ICA stenosis (Panel B)**.

In the case of bilateral ICA stenosis, we selectively removed the left or right ICA stenosis at each time. In this case, the types 4, 6, 8 and 9 CoW structures were found to induce post-CAS cerebral hyperperfusion (see Figure 8). Unlike the cases of the types 4, 6 structures, cerebral hyperperfusion induced by the types 8, 9 structures was not sensitive to the location side of the removed ICA stenosis and present in both hemispheres.

### Hyperperfusion rates in the case of coexisting BA stenosis and unilateral ICA stenosis

**Figure 9:**
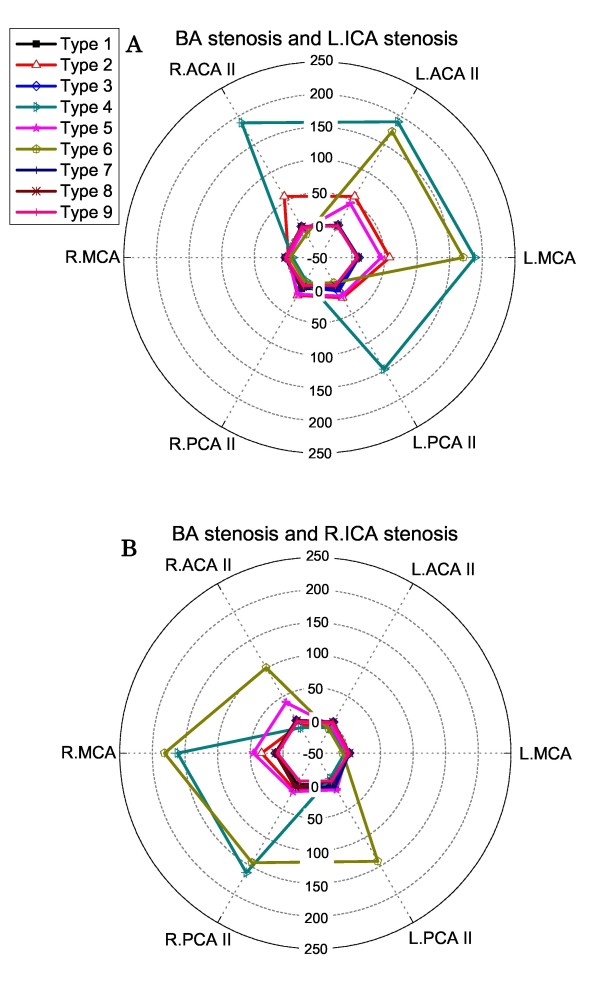
**Polar plots of the computed post-CAS hyperperfusion rates in the cerebral efferent arteries in the case of coexisting BA stenosis and unilateral ICA stenosis: left ICA stenosis (Panel A), right ICA stenosis (Panel B)**.

When BA stenosis existed concurrently with unilateral ICA stenosis, the types 4, 6 CoW structures that have been found to induce post-CAS hyperperfusion either in the case of left ICA stenosis or in the case of right ICA stenosis induced hyperperfusion in both cases (see Figure 9). Moreover, there was a significant enlargement of the cerebral region affected by post-CAS hyperperfusion (compare Figure 7 with Figure 9).

## Discussion

The pre-CAS status of cerebral hemodynamics has been found to be an important factor for assessing post-CAS cerebral hyperperfusion in patients with severe ICA stenosis [5,6,8]. Cerebral hemodynamics may be evaluated directly by measuring intra-arterial blood flow using magnetic resonance angiography or transcranial Doppler sonography [10,12,46,47] or indirectly via cerebral vasoreativity test [3], measurement of brain temperature [48] or brain oxygenation [49]. Despite the existence of these methods, an accurate measurement of blood flow rates in all the major cerebral arteries is yet difficult in clinical settings, which considerably hampers a full understanding of the collateral function of the cerebral artery network in pathological conditions. In contrast, the geometry of large cerebral arteries can nowadays be measured with satisfactory accuracy in clinical settings [11]. Computational hemodynamic modeling offers an alternative way to assess cerebral hemodynamics based on available geometrical data of cerebral arteries. A significant advantage of a computational model is that it allows us not only to quantify the blood flow rate in any cerebral artery of interest but also to evaluate the role of the entire cerebral artery network in regulating cerebral blood flows under various physiological/pathological conditions.

In this context, we have developed a 0-1D multi-scale model of the cerebral circulation (coupled with the global cardiovascular system) and applied it to investigate the influence of the anatomy of the CoW on post-CAS cerebral hyperperfusion. We should stress that although fully three-dimensional (3-D) modeling of the cerebral arteries can provide a more accurate and detailed description of blood flows compared to 0-D or 1-D modeling [45,50]; it is not practical for the present study due to its high computational cost. In this study, each set of computation has to be run continually for tens of cardiac cycles, for which reason a modeling method that incurs lower computational cost would be more favorable. Reducing 3-D modeling into 1-D modeling significantly reduces the required computational effort, but at the expense of the loss of some geometric information, such as local artery surface shape, curvature and bifurcation structure. There is evidence that computation results obtained with 1-D and 3-D models of the cerebral arterial network are in good agreement in terms of mass-flow distribution and pressure drop along arteries [50]. The purpose of the present study determines that we are interested in mass-flow distribution rather than in local flow patterns; therefore, 1-D modeling should be a choice with a good balance between computational demand and physical detail for the description of the cerebral arterial network. For the peripheral portion of the cerebral circulation, its extreme complexity determines that 0-D modeling is the only practical way. Particularly, 0-D modeling allows us to readily account for certain physiological or pathological conditions by modifying model parameters.

The computed results presented in Figure 7, 8, 9 indicate that (1) the anatomy of the CoW and the distribution pattern of stenosis in the cerebral feeding arteries jointly determine the risk and the localization of post-CAS cerebral hyperperfusion; (2) the existence of BA stenosis or contralateral ICA stenosis tends to increase the risk of post-CAS hyperperfusion and enlarge the cerebral region affected by hyperperfusion; and (3) some CoW structures may induce post-CAS hyperperfusion in both hemispheres under certain conditions, such as the types 8, 9 CoW structures combined with bilateral ICA stenosis.

### CoW structures susceptible to post-CAS hyperperfusion

As discussed above, the risk of a CoW structure for inducing post-CAS hyperperfusion should be always assessed in conjunction with the location of stenosis in the cerebral feeding arteries. According to the computed results, high-risk CoW structures are those (types 4 and 6) lacking collateral pathways from the contralateral ICA in the case of unilateral ICA stenosis and those lacking collateral pathways either from the contralateral ICA (types 4 and 6) or from the BA (types 8 and 9) in the case of bilateral ICA stenosis.

### Influences of the diameters of the cerebral communicating arteries and the severity of ICA stenosis on post-CAS hyperperfusion

**Figure 10:**
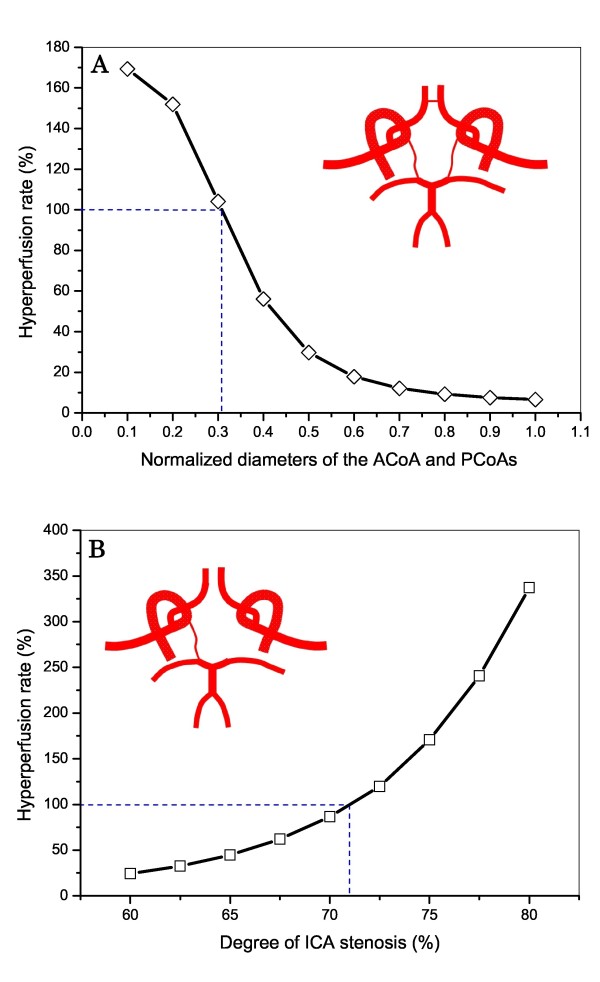
**Computed changes in hyperperfusion rate in the L**. MCA with changes in: (A) the diameters of the cerebral communicating arteries (type 1 CoW structure), and (B) the degree of the ICA stenosis (type 6 CoW structure). In both cases, the stenosis is located in the left ICA. The diameter values shown in the horizontal axis of Figure 10(A) are normalized by the default values of the communicating arteries.

Although the major objective of this study is to investigate the influence of the anatomy of the CoW on post-CAS hyperperfusion, we should point out that the diameters of the cerebral communicating arteries and the severity of cerebral feeding artery stenosis are important factors for assessing post-CAS hyperperfusion as well. To confirm this viewpoint, we additionally carried out two sets of computation. In both sets of computations, the stenosis was located in the left ICA. In the first set of computation, the diameters of the ACoA and PCoAs of the type 1 CoW structure were reduced step by step to 10% of their reference values; whereas in the second set of computation, the severity of the ICA stenosis was increased gradually from 60% to 80% (herein the type 6 CoW structure was studied). The computed post-CAS hyperperfusion rates in the left MCA for the two cases are plotted in panels A and B of Figure 10, respectively. It was observed that even the complete CoW structure may induce hyperperfusion if the communicating arteries are small, and the value of hyperperfusion rate is highly sensitive to the severity of the ICA stenosis, especially when the severity is greater than 70%.

### Sensitivity of cerebral arterial territory to post-CAS hyperperfusion

In the case of unilateral ICA stenosis, there is no apparent difference in hyperperfusion sensitivity between the ACA territory and the PCA territory; whereas, in the case of bilateral ICA stenosis, the ACA territory is more frequently affected by hyperperfusion in comparison with the PCA territory, with the appearance frequencies of hyperperfusion in these territories being 9 times vs. 3 times. The results support the clinical finding that a pronounced increase of blood flow velocity is often observed in the anterior part of the CoW immediately after CAS in the case of bilateral ICA stenosis [51]. This phenomenon can be explained from the fact that the PCA territory often possesses richer collateral pathways from the BA than the ACA territory, and the influence of this difference on cerebral perfusion is enhanced by the presence of bilateral ICA stenosis which reduces blood flows through both ICA, making cerebral perfusion rely more strongly on the blood flow supplied by the BA.

### Change in transmural pressure after CAS

The most pronounced changes in transmural pressure after CAS were observed in the microcirculations of the cerebral territories subjected to post-CAS hyperperfusion. For instance, the proximal (arteriolar limb) capillary pressure in the left MCA territory (type 6 CoW structure with L. ICA stenosis) increased from a pre-CAS value of 18.6 mmHg to a post-CAS value of 46.9 mmHg (see Figure 6(A)). This phenomenon is attributable to a reduction in arteriolar resistance under pre-CAS ischemic conditions, which leads to a shift of pressure distribution from the arterioles toward the capillaries, ultimately resulting in a high capillary pressure when the proximal arterial perfusion pressure is recovered after CAS. The prediction is consistent with the results of the ischemia-reperfusion experiments on the isolated dog hind limb [52]; whereas, whether the similar phenomenon occurs in vivo in the human cerebral circulation remains not well known. If it were true, it might augment capillary leakage, increase the risk of edema, and hence be another causative factor for post-CAS CHS in addition to increased blood flow rate [53].

## Limitations

A major limitation of this study is the absence of a sufficient comparison between model predictions and in vivo measurements. Actually, so far, we are not aware of any in vivo studies that systemically investigate the relationship between post-CAS hyperperfusion and the anatomy of the cerebral artery network with account of the distribution pattern of stenosis in the cerebral feeding arteries. At this point, further in vivo studies would be required to confirm the findings of the present study. Another limitation of this study may arise from the exclusion of the secondary cerebral collateral vessels from the present model, which potentially makes the model overestimate post-CAS hyperperfusion rate. Moreover, the CoW structures investigated in this study are limited to those illustrated in Figure 1(C), other CoW structures, such as those described elsewhere [14,18,44], would deserve further studies. Finally, since we did not take into account cerebral autoregulation in post-CAS computation by assuming cerebral distal resistances to remain constant after CAS, the predicted results may represent the largest values of post-CAS hyperperfusion rates. The dynamic cerebral autoregulation has been found to be significantly impaired in some patients with severe ICA stenoses [3,7,54]. After CAS, the immediate restoration of perfusion pressure does not guarantee an immediate sufficient restoration of cerebral autoregulation. In fact, hyperperfusion or impaired cerebrovascular reserve has been identified several days after CAS [3,7,55], indicating that cerebral autoregulation may remain insufficient for a fairly long time after CAS in some patients. In this sense, under in vivo conditions, the values of post-CAS hyperperfusion rates should be time-dependent, changing in close association with the post-CAS restoration of cerebral autoregulation. So far, the post-CAS restoring process of cerebral autoregulation remains not fully understood and seems to be strongly patient-specific [55], preventing us from developing a general model for describing post-CAS cerebral autoregulation. This limitation might be overcome if sufficient experimental data would be reported in the future.

Despite these limitations, some model-based findings regarding the factors tending to increase the risk of cerebral hyperperfusion (e.g., the existence of contralateral ICA stenosis, high-grade ICA stenosis) and the sensitivity of cerebral arterial territory to post-CAS hyperperfusion are in agreement with previous clinical findings [2,7,51]. Other model-based findings regarding the influences of BA stenosis and the diameters of the cerebral communicating arteries on the risk of post-CAS cerebral hyperperfusion and the change in capillary transmural pressure after CAS are reported for the first time. These findings, though awaiting further experiment-based confirmation, are of potential significance in the assessment and treatment of cerebral hyperperfusion.

## Conclusions

Using a computational model, this study demonstrated the importance of considering the anatomy of the CoW in assessing the risk of post-CAS cerebral hyperperfusion. Particularly, the finding that the anatomy of the CoW and the distribution pattern of stenosis in the cerebral feeding arteries jointly determine the risk and localization of post-CAS cerebral hyperperfusion suggests that a patient-specific hemodynamic analysis aimed to help physicians identify patients at high risk of post-operative cerebral hyperperfusion should account for the combined effect of the anatomy of cerebral arteries and cerebral feeding artery stenoses on cerebral hemodynamics.

## Competing interests

The authors declare that they have no competing interests.

## Authors' contributions

FYL designed the study, constructed the computational model and drafted the manuscript. KF put forward the issue from a clinical perspective and was involved in drafting the manuscript. HL analyzed the computed results and corrected the manuscript. ST supervised the whole process of the study and corrected the manuscript. All authors have read and approved the final manuscript.
